# Impact of smart healthcare-based behaviors of elderly patients with chronic diseases on physicians’ behavioral adaptations

**DOI:** 10.3389/fmed.2025.1595637

**Published:** 2025-08-13

**Authors:** Nan Ji, Mao Wu, Yong Liu

**Affiliations:** ^1^School of Design, Jiangnan University, Wuxi, China; ^2^Department of Orthopaedics, Wuxi Hospital of Traditional Chinese Medicine, Wuxi, China

**Keywords:** smart healthcare, elderly, chronic diseases, behavior, influencing factors

## Abstract

**Background:**

This study aimed to investigate how the smart healthcare-based behaviors of elderly patients with chronic diseases influence physicians’ behavioral adaptations.

**Methods:**

Physicians providing healthcare services to elderly patients with chronic diseases between July 1, 2024, and July 31, 2024, were recruited. A total of 100 physicians and 100 of their patients were enrolled. Data were collected using a general information questionnaire, the Chinese version of the Self-Efficacy in Patient-Centeredness Questionnaire (SEPCQ), the Chinese version of the Wake Forest Physician Trust Scale (WFPTS-C-10), the Health Information Seeking Behavior (HISB) scale, and the Cloud Follow-up Service Experience Scale for Patients with Chronic Diseases.

**Results:**

The mean scores were as follows: SEPCQ (50.54 ± 6.16), WFPTS-C-10 (107.82 ± 5.16), HISB (31.96 ± 4.94), and the Cloud Follow-up Service Experience Scale for Chronic Disease Patients (26.11 ± 3.16). No statistically significant differences were observed (*p* > 0.05). There were statistically significant differences in SEPCQ scores among physicians of different ages, frequencies of individual communication with patients per week and years of working experience (*p* < 0.05). Correlation analysis revealed that SEPCQ scores were positively correlated with the scores of WFPTS-C-10, HISB, age, number of individual communications with patients per week, and working years (*r* = 0.264, 0.289, 0.311, 0.276, 0.333, *p* < 0.001), and negatively correlated with the scores of Cloud Follow-up Service Experience Scale for Patients with Chronic Diseases (*r* = −0.879, *p* < 0.001). Multiple linear regression analysis showed that age, the number of separate communications with patients per week, working years, WFPTS-C-10, HISB and the scores of Cloud Follow-up Service Experience Scale for Patients with Chronic Diseases were significant predictors of SEPCQ scores (*p* < 0.05), accounting for 38.7% of the variance.

**Conclusion:**

In the current healthcare context, behaviors of elderly patients with chronic diseases significantly influence physicians’ behavioral adaptations.

## 1 Background

With the acceleration of population aging, health management for elderly patients with chronic diseases has become a major challenge for global healthcare systems (1). According to the Report on Chinese Residents’ Chronic Diseases and Nutrition 2015, chronic diseases have become the main cause of death among residents in China, especially cardiovascular and cerebrovascular diseases, cancers and chronic respiratory diseases. In this context, the rapid development of smart healthcare technologies and service design thinking in medical services have provided new solutions and ideas for chronic disease management (2, 3). Smart healthcare integrates advanced technologies such as artificial intelligence (AI), the Internet of Things (IoT), big data, and cloud computing, not only realizes real-time monitoring and analysis of patient health data, but also provides personalized health management solutions, effectively improving the efficiency and quality of medical services (4). For elderly patients with chronic diseases, the intervention of smart healthcare not only helps them to monitor their own health status more conveniently, but also promotes information communication and interaction between physicians and patients, making medical services more accurate and efficient (5–8). Meanwhile, with the advent of the experience economy era, the traditional service industry has been redefined. Customers no longer merely need a single service, but rather hope to obtain services that are useful, easy to use, and desirable, aiming to provide patients with a positive experience throughout the service process. Physician behavioral adaptation refers to the flexible adjustments physicians make in their medical practices, such as diagnostic strategies, treatment plans, and communication methods, based on patient-specific conditions, disease progression, and treatment efficacy. These adaptations aim to better meet patient needs, improve treatment outcomes, promote patient recovery, and ensure the personalization and efficiency of medical services. However, research on physician behavioral adaptation remains limited, and few studies have explored the impact of elderly patients with chronic diseases on physician behavioral adaptation within the context of smart healthcare. Therefore, this study aims to explore how the smart healthcare-based behaviors of elderly patients with chronic diseases influence physicians’ behavioral adaptations. By employing service design thinking to analyze user behavior and conducting an in-depth examination of the behavioral characteristics of elderly patients on smart healthcare platforms, this research seeks to elucidate the practical applications of smart healthcare in chronic disease management. The findings will provide a theoretical foundation and practical insights for optimizing medical service models and enhancing patient health management.

## 2 Methods

### 2.1 Research objects

Physicians specializing in geriatric chronic diseases between July 1, 2024, and July 31, 2024, were recruited as participants. Physicians were eligible to participate if they (1) possessed a valid medical license (2), held a bachelor’s degree or higher (3), had been employed at the hospital for at least 3 months (4), served as the attending physician for patients with chronic diseases, and (5) voluntarily agreed to participate with signed informed consent. Physicians were excluded if they (1) were undergoing standardized training (2), were absent from the hospital due to study or other reasons during the investigation period, or (3) withdrew from the study for personal reasons. All participants provided signed informed consent, and the study was approved by the Medical Ethics Committee of the hospital.

### 2.2 Patient inclusion and exclusion criteria

Inclusion criteria: (1) patients who met the clinical diagnosis of any chronic disease (2); patients aged ≥60 years; and (3) patients who voluntarily participated and provided signed informed consent. Exclusion criteria: (1) patients whose condition was life-threatening; and (2) patients with mental disorders.

#### 2.2.1 Sample size calculation

According to established guidelines for factor analysis studies, the sample size should typically be 5 to 10 times the number of variables included (9, 10). This study involved 13 variables. Considering a potential 20% invalid response rate, the required sample size ranged from 78 to 156. Ultimately, 100 physicians were enrolled using a random sampling method. A total of 100 patients diagnosed and treated by the participating physicians were randomly selected. These patients included 36 with cardiovascular and cerebrovascular diseases, 24 with chronic respiratory diseases, 19 with diabetes, and 21 with hypertension.

#### 2.2.2 Participant demographics and bias control measures

To address potential biases, this study employed random sampling to ensure sample representativeness, utilized multivariate analysis to control confounding variables (e.g., age and gender), and implemented blinded evaluations to minimize subjective bias. These measures ensured the accuracy and reliability of the results while effectively mitigating the impact of biases and confounding factors.

#### 2.2.3 General data

This survey was conducted through a questionnaire independently designed by the investigator. The contents of the questionnaire included 9 items, including gender, age, marital status, working years, professional title, administrative position, establishment, average daily working hours and the number of separate communications with patients per week.

### 2.3 Chinese version of Self-Efficacy in Patient-Centeredness Questionnaire

The Chinese version of the Self-Efficacy in Patient-Centeredness Questionnaire (SEPCQ), developed by Dongxue et al. (11), was utilized. This scale comprises three dimensions: identifying patient needs (9 items), sharing information and power (10 items), and addressing communication challenges (7 items), totaling 26 items. Each item is scored on a 5-point Likert scale ranging from 0 to 4, with higher scores indicating greater self-efficacy. The overall Cronbach’s *α* coefficient of the scale was 0.988, and Guttman’s split-half coefficient was 0.961, indicating that the scale has good reliability.

### 2.4 Chinese version of the Wake Forest Physician Trust Scale

The Wake Forest Physician Trust Scale (WFPTS-C-10) (12) is used to evaluate the patients’ trust in the physicians, including 10 items with 1–5 points for each item. Among them, 3 items (2, 3, 8) are scored reversely, and the total score of the scale is 1–50 points. For comparison, the original score can be converted into 0–100 points. The higher the total score, the higher the patient’s trust in the physician. The overall Cronbach’s alpha coefficient of the scale is 0.900, and the Guttman split-half coefficient is 0.87, indicating that the scale has good reliability.

### 2.5 Health Information Seeking Behavior

The Chinese Health Information Seeking Behavior (HISB) scale of Qiuzi et al. (13) was adopted, including 4 dimensions: health information seeking attitude (6 items), information needs (14 items), information sources (15 items) and barriers to obtaining health information (8 items). There were 43 items in total, with 1–5 points for each item. Higher scores indicate higher health information-seeking behaviors. The Cronbach’s alpha coefficient of the scale is 0.90, indicating that the scale has good reliability.

### 2.6 Cloud Follow-up Service Experience Scale for Patients with Chronic Diseases

The Cloud Follow-up Service Experience Scale for Patients with Chronic Diseases developed by Suang et al. (14) is adopted, including 5 dimensions of tangibility, reliability, responsiveness, assurance and empathy, with a total of 37 items. Each item is scored from two aspects: service expectation (“very low to very high,” scoring 1–7 points) and service feeling (“very poor to very good,” scoring 1–7 points), and the service feeling-service expectation is calculated. The difference is used as an evaluation criterion. A smaller difference indicates a better service experience. The Cronbach’s alpha coefficient of this scale is 0.962, indicating that the scale has good credibility.

### 2.7 Survey methods

All investigators underwent standardized training prior to data collection to ensure consistency in administering the survey. Questionnaires were distributed to physicians and patients during appropriate clinical hours when participants had sufficient time. Before distribution, the purpose, significance, and instructions for completing the questionnaire were explained in detail. Written informed consent was obtained from all participants. During completion, any questions raised by participants were addressed on-site by the research team. After collection, all questionnaires were reviewed for completeness and accuracy. Invalid or incomplete responses were excluded from analysis. In total, 111 questionnaires were distributed, and 100 valid responses were collected, yielding an effective response rate of 90.09%.

### 2.8 Statistical analysis

After the questionnaire collection was checked by two persons, the data were entered, analyzed, and processed with SPSS 23.0. Measurement data were expressed as (mean ± standard deviation) when they obeyed normal distribution, and independent sample *t*-test was used for comparison between two groups; F-test was used for multiple group comparison, enumeration data were expressed as “*n*/%,” and chi-square test was used for comparison. Those with statistical significance in univariate analysis were included in multivariate analysis. Logistic regression model was used for multivariate analysis, and *p* < 0.05 indicated that the difference was statistically significant.

## 3 Results

### 3.1 Scale scores

**Figure 1:**
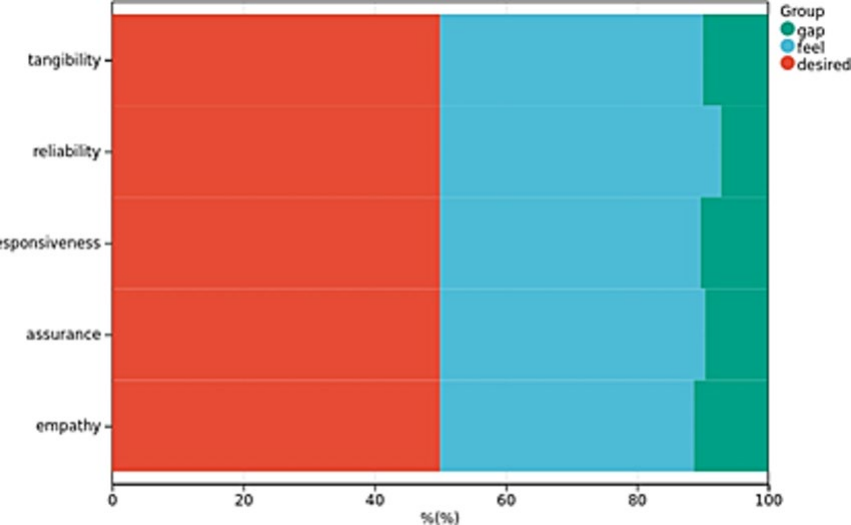
Patient Experience Scores for Cloud-Based Follow-Up Services in Chronic Disease Management. The figure highlights a significant discrepancy between patients’ actual experiences and their initial expectations regarding the cloud follow-up services.

The mean total scores were as follows: SEPCQ (50.54 ± 6.16), WFPTS-C-10 (107.82 ± 5.16), HISB (31.96 ± 4.94), and the Cloud Follow-up Service Experience Scale for Patients with Chronic Diseases (26.11 ± 3.16), as presented in Tables 1–3 and Figure 1.

**Table 1:** Scoring of SEPCQ.

Dimensions	Mean score (points)
Identify patient needs	15.54 ± 2.14
Sharing information and authority	20.64 ± 2.33
Addressing communication challenges	14.36 ± 2.64

**Table 2:** Scoring of WFPTS-C-10.

Factor 1 distrust		Factor 2 distrust	
Item	Average score of items (points)	Item	Average score of items (points)
2	3.11 ± 0.95	1	3.19 ± 0.33
3	3.46 ± 0.74	4	2.11 ± 0.21
8	3.79 ± 0.64	5	3.55 ± 0.34
		6	3.43 ± 0.37
		7	3.13 ± 0.52
		9	3.22 ± 0.41
		10	2.97 ± 0.33

**Table 3:** HISB score.

Dimensions	Mean score (points)
Health information seeking attitudes	15.48 ± 2.64
Information requirements	34.64 ± 2.56
Source of information	37.54 ± 2.82
Barriers to accessing health information	20.16 ± 2.94

### 3.2 Comparative analysis of SEPCQ scores by physician characteristics

No significant differences in SEPCQ scores were observed based on gender, marital status, professional title, administrative position, employment status, or average daily working hours (*p* > 0.05). However, significant differences were found among physicians of different age groups (≥45 years vs. <25 and 25–45 years), frequency of individual patient communications per week (>3 times vs. ≤3 times), and years of work experience (>5 years vs. ≤3 and 3–5 years) (*p* < 0.05) (Table 4).

**Table 4:** General data survey results (*n* = 100).

Item		Number of cases	SEPCQ scale (score)	*t*/*F* value	*p*-value
Sex (*t*-test)	Male	55	50.43 ± 5.73	0.194	0.847
	Female	45	50.64 ± 4.94		
Age (years) (*F*-test)	<25	25	47.64 ± 4.64	29.632	<0.001
	25–45	43	52.64 ± 5.17		
	≥45	57	56.64 ± 4.91		
Marital status (*t*-test)	Married	72	50.94 ± 4.72	0.170	0.866
	Single	28	50.76 ± 4.88		
Years of working (*F*-test)	≤3 years	25	45.39 ± 4.55	33.072	<0.001
	3–5 years	23	50.94 ± 5.17		
	>5 years	52	55.64 ± 5.54		
Title (*F*-test)	N/A	12	49.64 ± 3.94	1.037	0.392
	Primary	5	50.28 ± 4.22		
	Intermediate	46	51.11 ± 3.62		
	Associate	27	51.97 ± 4.21		
	Senior	10	52.34 ± 3.96		
Administrative Positions (*t*-test)	Yes	12	50.77 ± 4.31	0.623	0.535
	N/A	88	51.64 ± 4.57		
Institution (*t*-test)	Staff registered with the institution	41	51.33 ± 3.84	0.849	0.398
	Staff do not register with the institution	59	51.97 ± 3.61		
Average daily working hours (*F*-test)	≤8 h	7	49.94 ± 4.55	0.039	0.990
	8–10 h	67	50.43 ± 5.65		
	10-12 h	21	50.21 ± 4.49		
	>12 h	5	50.87 ± 5.57		
Number of separate communications with patients per week (*t*-test)	≤3 times	37	48.49 ± 4.92	3.433	0.001
	>3 times	63	52.33 ± 5.66		

### 3.3 Correlation analysis between SEPCQ and other scales

Correlation analysis revealed that SEPCQ scores were positively correlated with WFPTS-C-10 scores, HISB scores, age, frequency of individual patient communications per week, and years of work experience (*r* = 0.264, 0.289, 0.311, 0.276, 0.333, respectively; *p* < 0.001), and negatively correlated with the Cloud Follow-up Service Experience Scale for Patients with Chronic Diseases (*r* = −0.879, *p* < 0.001) (see Table 5).

**Table 5:** Correlation analysis between SEPCQ and other scales.

Scale	*r*	*p*
WFPTS-C-10	0.264	<0.001
HISB	0.289	<0.001
Cloud Follow-up Service Experience Scale for Patients with Chronic Diseases	−0.879	<0.001
Age	0.311	<0.001
Number of individual communications with patients per week	0.276	<0.001
Working years	0.333	<0.001

### 3.4 Multiple linear regression analysis

Incorporate meaningful variables from single factor analysis and correlation analysis into multiple linear regression analysis. See Table 6 for the assignment method of variables. Multiple linear regression analysis indicated that age, frequency of individual patient communications per week, years of work experience, WFPTS-C-10 scores, HISB scores, and Cloud Follow-up Service Experience Scale scores were significant predictors of SEPCQ scores (*p* < 0.05), accounting for 38.7% of the variance (Table 7).

**Table 6:** Assignment of variables.

Variable	Assignment method
Age	<25 = 1, 25–45 = 2, ≥45 = 3
Number of separate communications with patients per week	≤3 times = 1, >3 times = 3
Years of working	≤3 years = 1, 3–5 years = 2, >5 years = 3
WFPTS-C-10	Brought in by original value
HISB	Brought in by original value
Cloud Follow-up Service Experience Scale for Patients with Chronic Diseases	Brought in by original value

**Table 7:** Multiple linear regression analysis (*n* = 100).

Item	*β*	SE	*β*′	*t*	*p*
Age	1.074	0.521	0.161	2.791	<0.001
Number of separate communications with patients per week	0.953	0.354	0.297	2.942	<0.001
Years of working	0.946	0.344	0.264	2.772	<0.001
WFPTS-C-10	0.897	0.294	0.278	3.194	<0.001
HISB	1.064	0.465	0.154	2.348	0.024
Cloud Follow-up Service Experience Scale for Patients with Chronic Diseases	−1.573	0.431	−0.344	−3.761	<0.001

*R*^2^ = 0.392, adjusted *R*^2^ = 0.387, *F* = 8.721, *p* < 0.001.

## 4 Discussion

The effective management of chronic diseases in elderly patients remains a significant challenge for global healthcare systems. The emergence of smart healthcare, characterized by its convenience, efficiency, and personalization, offers novel solutions for the long-term management of chronic conditions in this population. However, this transformation requires not only technological innovation but also profound behavioral adaptations from both physicians and patients. From a service design perspective, the behaviors of participants in the service process not only affect service quality and effectiveness but also influence the experiences of service recipients and the work quality of service providers. By comprehensively analyzing how the behaviors of elderly patients with chronic diseases influence physicians’ behavioral adaptations, this study aims to explore a new model of physician-patient interaction within the smart healthcare context and to provide theoretical support and practical guidance for optimizing medical services, enhancing patient satisfaction, and improving medical service outcomes (15). However, this transformation necessitates not only technological innovation but also profound adaptations in the behaviors of both physicians and patients. From a service design perspective, the behaviors of participants in the service process not only affect service quality and effectiveness but also influence the experiences of service recipients and the work quality of service providers. By comprehensively analyzing how the behaviors of elderly patients with chronic diseases influence physicians’ behavioral adaptations, this study aims to explore a new model of physician-patient interaction within the smart healthcare context and to provide theoretical support and practical guidance for optimizing medical services, enhancing patient satisfaction, and improving medical service outcomes.

### 4.1 Scale selection and smart medicine discussion

In this study, four strictly validated scales targeting specific target populations were selected to comprehensively assess patients’ multi-faceted experiences in the context of smart medicine. First, the SEPCQ scale is mainly aimed at patients participating in medical services and aims to explore their ability to identify their own needs, share information with medical staff, and respond to communication challenges in a smart medical environment. This scale was chosen because it can reflect patients’ self-efficacy under new medical models and is crucial to optimizing patient participation and satisfaction. WFPTS-C-10 is widely used to assess patients’ trust in doctors, especially in the context of smart medicine, where trust is the basis for establishing a good doctor-patient relationship. The scale measures trust through multiple dimensions and helps understand how trust affects patients’ medical decisions and compliance behaviors. HISB is aimed at all individuals who are concerned about their own health and proactively seek health information. In the era of smart healthcare, information acquisition is particularly important. This scale can assess individuals’ attitudes, needs, sources and obstacles in seeking health information, and provide a basis for providing customized information services. The Chronic Disease Patient Cloud Follow-up Service Experience Scale is specially designed for patients with chronic diseases and aims to assess their experience while receiving cloud follow-up services. With the development of smart healthcare, cloud follow-up has become an important means of chronic disease management. This scale comprehensively evaluates the service experience from multiple dimensions, which helps identify shortcomings in the service and continuously improve it. These scales were selected not only because they have good reliability and validity, but also because they can highlight the core needs of patients in terms of self-management, trust building, information acquisition and service experience in the context of smart medicine, and improve the quality and efficiency of medical services. Provide important reference.

### 4.2 Behavioral characteristics in elderly chronic disease patients

#### 4.2.1 Positive information-seeking behavior

The results of HISB scale show that elderly patients with chronic diseases generally exhibit higher health information-seeking behaviors (total score of HISB: 31.96 ± 4.94), which reflects that in the era of information explosion, patients pay more and more attention to self-health management and knowledge acquisition. They obtained disease knowledge, medical service plans and prevention strategies through various channels such as the Internet, social media and health APPs, which to some extent promoted the improvement of information asymmetry between physicians and patients (16). However, it also places greater demands on physicians to have stronger professional qualities and communication skills to guide patients in screening, understanding and applying the right health information.

#### 4.2.2 Differentiated performance of trust in physicians

The WFPTS-C-10 scale score (107.82 ± 5.16 points) showed that most patients had a high degree of trust in physicians. This trust is the foundation of the physician-patient relationship and one of the key factors for successful medical service. However, it is worth noting that the establishment and maintenance of patient trust are not achieved overnight. It is affected by various factors such as physicians’ professional competence, service attitude, communication skills, patients’ personal experiences and cultural background (17). In the context of smart healthcare, patients may communicate with physicians through online platforms, and this non-face-to-face communication may pose new challenges for trust building (18, 19). Therefore, physicians need to pay more attention to the art of communication and enhance patients’ trust through timely, accurate and personalized responses.

#### 4.2.3 Experience and feedback of cloud follow-up service

The score of the Cloud Follow-up Service Experience Scale for Patients with Chronic Diseases (26.11 ± 3.16 points) is relatively low, reflecting that there are still some problems and deficiencies in the practical application of cloud follow-up service at present. This may be related to factors such as system operation complexity, information security and service response speed. Negative experiences of patients may reduce their acceptance and satisfaction with smart healthcare services, which in turn affects their trust in physicians and medical service adherence (20). Therefore, when promoting smart healthcare services, medical institutions should fully consider the actual needs and use experience of patients, continuously optimize service processes and technical support, and enhance patient satisfaction and loyalty.

#### 4.2.4 Influencing factors of physician behavior adjustment

The study found that age and working years are important factors affecting physicians’ “patient-centered” self-efficacy (SEPCQ score). Young physicians may be unable to deal with complex conditions and patients’ needs due to inexperience, while senior physicians are more likely to establish a trust relationship with patients and realize the patient-centered medical service concept by virtue of rich clinical experience and profound humanistic care (21). This suggests that during the promotion of smart healthcare, attention should be paid to training and guiding young physicians to help them grow rapidly and adapt to the new medical model. The number of separate communications with patients per week had a significant impact on SEPCQ scores, indicating that effective communication was the key to establishing a good physician-patient relationship. Although smart healthcare provides convenient communication channels, face-to-face communication still plays an irreplaceable role in building trust, conveying emotions and resolving doubts (22). Therefore, physicians should arrange their time reasonably, increase the frequency of communication with patients and improve the quality of communication to better meet the psychological needs and medical service expectations of patients.

#### 4.2.5 Patient behaviors and trust

SEPCQ score was positively correlated with WFPTS-C-10 and HISB scores, indicating that patients’ trust and information-seeking behavior had a positive impact on physicians’ behavior adjustment. Patients’ trust can stimulate physicians’ enthusiasm and sense of responsibility for work, prompting them to pay more attention to patients’ needs and feelings; while patients’ positive information-seeking behavior helps physicians understand the cognitive level and psychological state of patients, to formulate a more personalized and targeted medical service plan (23). Therefore, physicians should encourage patients to participate in the process of obtaining health information and decision-making, while strengthening their professionalism and communication skills to win patients’ trust and respect.

### 4.3 Improvement measures

First of all, improving patient self-efficacy and participation is one of the core. Research results show that doctors with higher SEPCQ scores are more likely to be influenced by patient behavior and make positive adjustments. Therefore, it is necessary to carry out patient education programs, such as chronic disease self-management courses, to enhance patients’ disease management capabilities and self-confidence. These courses can cover basic knowledge of diseases, lifestyle adjustments, drug treatment and other aspects to help patients better understand and cope with their diseases. At the same time, encouraging patients to use smart medical platforms, such as mobile health apps, for self-monitoring and management is also an effective way to improve their participation and self-efficacy. These platforms can provide real-time data monitoring, health reminders, medication guidance and other functions to help patients better manage their health conditions. Secondly, optimizing doctor-patient communication is the key to improving the quality of medical services. The study found that doctors who communicated more individually with patients per week also had higher SEPCQ scores. This shows that frequent doctor-patient communication helps doctors better understand patient needs and make adjustments accordingly. Therefore, medical institutions should encourage doctors to increase the time they spend face-to-face or online communication with patients, especially when using smart medical platforms for remote consultation, to ensure the quality and depth of communication. In addition, training doctors to improve communication skills so that they can more effectively listen to patients’ opinions and understand patients’ needs is also an important means to enhance the trust relationship between doctors and patients. In terms of improving doctors’ sensitivity and adaptability to patients’ behavior, it is necessary to improve doctors’ ability to identify patients’ behavior and mental state through regular training and case sharing. In this way, doctors can better adjust treatment plans and communication strategies to adapt to changing behaviors and needs of patients. At the same time, doctors should also be aware of the impact of patient behavior on their medical decisions and learn to respond flexibly in medical practice. Although research results show that there is a negative correlation between the experience of cloud follow-up services for patients with chronic diseases and doctors’ self-efficacy, this does not mean that we should give up the application of smart medical technology. Instead, we should further optimize the functions and user experience of cloud follow-up services, such as providing more personalized health advice, more convenient consultation channels, etc., to enhance patient satisfaction and participation. At the same time, collecting patient feedback and continuously improving the design and functions of smart medical platforms are also important ways to meet patient needs and improve the quality of medical services. In addition, it is also necessary to develop personalized interventions based on different characteristics of patients (such as age, disease course, etc.). For older patients, more patience and careful explanation may be needed to ensure they can understand and follow the treatment plan. For patients with long courses of disease, more comprehensive health management and psychological support are needed to reduce their disease burden and psychological stress. These personalized interventions can help patients better cope with disease challenges and improve their quality of life.

Finally, strengthening doctor-patient cooperation and team building is also a key link in improving the quality of medical services. Establish a multidisciplinary team collaboration mechanism, including doctors, nurses, psychological counselors, etc., to jointly provide comprehensive services to patients. Through team building activities, enhance communication and collaboration capabilities among medical staff to better respond to the changing needs of patients. This teamwork model ensures that patients receive comprehensive and professional support when receiving medical services.

### 4.4 Research innovations and deficiencies

This study is the first to investigate the impact of elderly patients with chronic diseases on physicians’ behavioral adaptations within the context of smart healthcare. From a research perspective, we break through the limitations of traditional doctor-patient relationship research, incorporate the emerging element of smart medicine into it, and deeply analyze how patient behavior affects doctors in digital medical scenarios. In terms of research methods, multiple scales are comprehensively used to survey doctors and patients to obtain data comprehensively and accurately. Through multiple linear regression analysis, multiple factors that affect doctors’ “patient-centered” self-efficacy are identified, providing a new perspective for understanding the doctor-patient interaction mechanism, and also providing a theoretical basis for subsequent optimization of medical services and improvement of doctors’ adaptability. Although this study shows that the behavior of elderly patients with chronic diseases has an impact on the adjustment of doctors’ behavior, there are also deficiencies. First of all, there is doctor participation in this study, which is most likely to introduce bias, especially when doctors with specific characteristics (such as being proficient in technology and being patient centered) are more inclined to participate. In order to make up for this shortcoming, multi-source data collection should be adopted in the future, patient self-reporting should be increased, and doctor characteristics should be considered as analysis variables to comprehensively evaluate the actual effect of smart medicine.

### 4.5 Future prospects

Based on this study’s finding that the behavior of elderly patients with chronic diseases affects the adjustment of doctors’ behavior, there are broad research prospects on doctors’ proactive adaptation strategies in the future. On the one hand, we can explore in depth how doctors use smart medical technology to proactively perceive changes in patient behavior. For example, with the help of patient health data collected by wearable devices and patient feedback information on online communication platforms, we can predict patient needs in advance and proactively adjust communication methods and diagnosis and treatment plans. On the other hand, we studied the differences in active adaptation strategies among doctors with different working years, ages and other characteristics. Younger doctors may be more skilled in the application of technology, while older doctors have more clinical experience and understand the impact of different strategies on patients. We can explore how to combine the advantages of both to form more effective adaptation strategies. In addition, attention can also be paid to the impact of physician active adaptation strategies on patients’ long-term health management effects, such as disease recurrence rate, quality of life, etc. Through these studies, scientific and practical active adaptation strategies are provided for doctors, the quality of medical services is improved, doctor-patient harmony is promoted, and the in-depth application of intelligent medical care in the management of chronic diseases in the elderly is promoted.

## 5 Conclusion

In conclusion, the behaviors of elderly patients with chronic diseases significantly influence physicians’ behavioral adaptations. Within the smart healthcare context, physicians must pay greater attention to patients’ information needs, trust-building, and psychological state changes to effectively meet their diverse needs through enhanced communication and personalized medical service plans. Concurrently, medical institutions should increase investment and support for smart healthcare services, continuously optimize service processes and technical support systems, and improve both patient satisfaction and medical service outcomes. Future research should further explore novel mechanisms and models of physician-patient interaction within the smart healthcare context, investigating various approaches to integrate service design thinking into medical service delivery. This will provide robust support for fostering harmonious physician-patient relationships and promoting the high-quality development of healthcare services.

## Data Availability

The raw data supporting the conclusions of this article will be made available by the authors, without undue reservation.
